# Does Modification to the Approach to Contacting Potential Participants Improve Recruitment to Clinical Trials?

**DOI:** 10.14740/jocmr1879w

**Published:** 2014-07-28

**Authors:** Vinit Sawhney, Adam Graham, Niall Campbell, Richard Schilling

**Affiliations:** a Department of Cardiology, St Bartholomew’s Hospital, Queen Mary University of London, UK

**Keywords:** Clinical trials, Patient recruitment, Telephone call

## Abstract

**Background:**

It is critical that clinical trial researchers ensure efficient and successful patient recruitment. Recruitment is often slower than expected and required sample sizes not obtained within initial funding deadlines. There is little rigorous evidence supporting ways to improve recruitment. We hypothesized making telephone contact with subjects prior to hospital attendance would improve recruitment rates into clinical trials.

**Methods:**

Retrospective *post hoc* analysis of recruitment rates in an on-going clinical trial was undertaken. Two hundred twelve consecutive patients were recruited over 6 months. During the first 3 months, patients received a telephone call from the research team and also received an information sheet by post prior to clinic attendance (group 1). The study was discussed on telephone and any issues were re-addressed at the patient’s clinic appointment when they were formally invited to participate in the study. After 3 months, the investigators stopped telephoning the patients (group 2); patients were invited to participate in the study by post and were first spoken to directly by an investigator in clinic. The study protocol and investigators did not change between groups.

**Results:**

There was no significant difference in baseline demographics between the two groups. There was a significant improvement in recruitment rate in group 1 compared to group 2 (77.7% vs. 45.0%, P < 0.0001). An improvement in clinic attendance rate in group 1 was observed, although this was not significant (did not attend rate: 2.9% vs. 7.8%, P = 0.14).

**Conclusion:**

Telephone contact between researchers and potential participants prior to clinic attendance can greatly improve study recruitment rates. This information may benefit the design of all clinical studies.

## Introduction

Recruitment of patients to research studies can be an immense challenge to investigators conducting large-scale clinical trials. Recruitment can be viewed as a surrogate measure of other, less easily quantifiable, but arguably more significant measures of trial success, such as “impact on clinical practice” or the extent to which the trial question has been answered. In the UK, in a cohort of 114 multicenter trials funded by the UK Medical Research Council and the UK Health Technology Assessment Program between 1994 and 2002, less than one-third (31%) of the trials achieved their original recruitment target and more than half (53%) were awarded an extension [1].

Failure to achieve efficient and successful participant recruitment can lead to increased trial costs, underpowered studies leading to potentially false results, delayed reporting of results or abandoning of studies altogether [2].

Factors that have been associated with successful recruitment included one or more interventions available only inside the trial, presence of a dedicated trials manager, cancer/drug trials and the use of newsletters/mailshots. However, these have not been causally linked to changes or differences in recruitment [3].

There is little rigorous evidence supporting approaches to improve recruitment and the relative effectiveness of different methods is still uncertain.

We hypothesized that improving communication with potential participants by making telephone contact with them prior to hospital attendance, in addition to posting patient information sheets, would improve recruitment rates into clinical trials.

## Methods

An observational cohort study of defibrillator patients attending the cardiac arrhythmia clinic at St Bartholomew’s Hospital was being performed which had no long-term follow-up requirements.

A *post hoc* retrospective analysis of recruitment rates in this study was undertaken. During the first 3 months of recruitment, the patients received a telephone call from the research team and received a patient information sheet by post prior to clinic attendance (group 1). During the telephone consultation, 1) the patients were invited to take part in the research trial, 2) the purpose and protocol of the study were explained and 3) any resulting questions were answered. Typically a telephone call lasted 15 - 20 min and the patients were also given a contact number for future reference. These issues were re-addressed at the patient’s clinic appointment when they were formally invited to participate in the research study.

After 3 months, the investigators stopped telephoning the patients (group 2). In this population, patients were invited to participate in the research trial by post. They were first spoken to by a study investigator in clinic when they were formally invited to participate. The study protocol and investigators did not otherwise change between the groups.

Demographic factors which might influence recruitment rates were collated for both groups.

Statistical analysis was performed using GraphPad Prism 5. Continuous data were compared using unpaired *t* test, whereas categorical data were compared by Chi-square test. A probability value of P < 0.05 was defined as significant.

## Results

Two hundred twelve consecutive patients with defibrillator implants attending the cardiac arrhythmia clinic at St Bartholomew’s Hospital, over a 6-month period were included. All patients were sent information about the research trial 3 weeks prior to their clinic appointment. Patients recruited over the first 3 months received a telephone call from the investigators prior to clinic attendance (group 1). Those recruited over the final 3 months were not contacted prior to clinic attendance (group 2). Table 1 demonstrates that there were no differences between the groups in terms of baseline demographic data.

**Table 1:** Patient Characteristics

	Group 1 (telephoned)	Group 2 (not telephoned)	P value
Number of patients	103	109	
Age, mean (range)	70.2 (46 - 87)	72.1 (40 - 88)	0.17
Female gender (%)	14.4	13.8	1
Distance from hospital (miles), mean (SEM)	18.9 (1.7)	21.5 (2.5)	0.39
Time since implant (months), mean (SEM)	38.5 (3.0)	54.9 (13.3)	0.25

**Figure 1:**
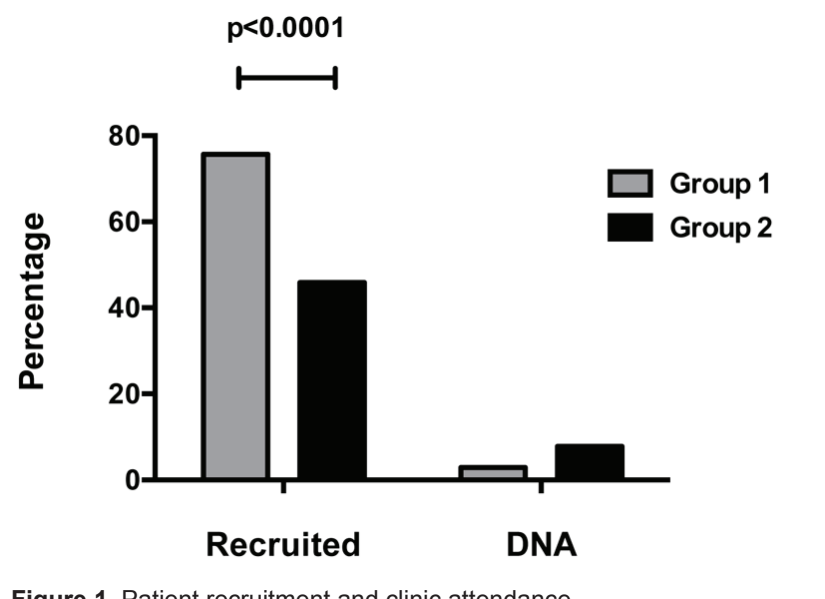
Patient recruitment and clinic attendance.

There was a significant improvement in recruitment rate in group 1 compared to group 2 (77.7% vs. 45.0%, RR: 1.72, P < 0.0001) as shown in Fig. 1.

An improvement in clinic attendance rate in group 1 was observed, although this was not significant when compared to the other group (did not attend rate: 2.9% vs. 7.8%, P = 0.14).

## Discussion

To the best of our knowledge, this is the first study to demonstrate that initial telephone contact between clinical trial researchers and potential participants prior to hospital attendance can improve recruitment rates.

Recruitment rates to clinical studies are frequently low and simple approaches to improve recruitment efficiency are needed. Low and uneven recruitment results in demoralized and inconvenienced staff and participants, excessive workload and reduced scientific value of the results by potential introduction of bias in underpowered studies [4].

In this study, all patients across the entire cohort were sent information about the research trial 3 weeks prior to hospital attendance. However, in group 1, the patients received a telephone consultation with the research team before they were invited to participate in the study. The average time of the telephone call was 10 - 15 min which gave the patients an opportunity to ask any research related questions. When they were subsequently seen in clinic, formal written consent was obtained. The average time spent with the patients was not significantly different between the groups. The two groups were well matched for other potential confounding factors including age, gender, travel distance (from hospital) and average duration of disease process. Not only were the recruitment rates better in group 1, but the sizeable difference (77% vs. 45%, P < 0.0001) reflects the impact of a simple intervention (telephone call) on improving recruitment rates.

The very low recruitment rate in group 2 (< 50%) provides a benchmark proportion for study recruitment in clinical research trials where patient commitment and interventions (a single blood test) would be considered to be low.

Suboptimal recruitment or lack of recruitment to any clinical trial can be due to various factors at different levels: the patient, the doctor, participating department, study organisation and design. Frequently mentioned barriers for patients include preference for one form of treatment, concerns with trial setting, general discomfort with research process, distrust in researchers, complexity of the protocol, potential side effect, fear that trial involvement would have a negative effect on the relationship with their physician, the potential for increased demands and the mere inability to make a decision [5-7].

Majority of these can be tackled by better communication between the researcher and patient which gives the latter a chance to express their concerns and alleviate their doubts about the research trial and makes them more involved and in control of the entire process. This can be easily done by adapting a modified approach to contacting potential participants as shown in this study. The time and resources spent on this intervention are minimal and cost-effective, keeping in mind its massive effect on the primary recruitment aim.

### Conclusion

Telephone contact between researchers and potential participants prior to clinic attendance can greatly improve study recruitment rates and is cost-effective from a staff resource perspective. This information may benefit the design of all clinical studies.
